# Impact of high-pressure processing on the bioactive compounds of milk - A comprehensive review

**DOI:** 10.1007/s13197-024-05938-w

**Published:** 2024-03-06

**Authors:** Shahida Anusha Siddiqui, Sipper Khan, Nur Alim Bahmid, Andrey Ashotovich Nagdalian, Seid Mahdi Jafari, Roberto Castro-Muñoz

**Affiliations:** 1 Campus Straubing for Biotechnology and Sustainability, Technical University of Munich, Essigberg 3, 94315 Straubing, Germany; 2 German Institute of Food Technologies (DIL E.V.), Prof.-Von-Klitzing-Straße 7, 49610 Quakenbrück, Germany; 3 Institute of Agricultural Engineering, Tropics and Subtropics Group, University of Hohenheim, Stuttgart, Germany; 4 Research Center for Food Technology and Processing, National Research and Innovation Agency (BRIN), 55961 Yogyakarta, Indonesia; 5 North-Caucasus Federal University, Pushkina Street 1, 355009 Stavropol, Russia; 6 Faculty of Food Science and Technology, Gorgan University of Agricultural Sciences and Natural Resources, Gorgan, Iran; 7 Iran Food and Drug Administration, Halal Research Center of IRI, Ministry of Health and Medical Education, Tehran, Iran; 8 Faculty of Civil and Environmental Engineering, Department of Sanitary Engineering, Gdansk University of Technology, G. Narutowicza St. 11/12, 80–233 Gdansk, Poland

**Keywords:** High-pressure processing, Casein, Whey proteins, Lipid constituents, Bioactive compounds, Milk protein allergenicity

## Abstract

High-pressure processing (HPP) is a promising alternative to thermal pasteurization. Recent studies highlighted the effectivity of HPP (400–600 MPa and exposure times of 1–5 min) in reducing pathogenic microflora for up to 5 logs. Analysis of modern scientific sources has shown that pressure affects the main components of milk including fat globules, lactose, casein micelles. The behavior of whey proteins under HPP is very important for milk and dairy products. HPP can cause significant changes in the quaternary (> 150 MPa) and tertiary (> 200 MPa) protein structures. At pressures > 400 MPa, they dissolve in the following order: αs_2_-casein, αs_1_-casein, k-casein, and β-casein. A similar trend is observed in the processing of whey proteins. HPP can affect the rate of milk fat adhering as cream with increased results at 100–250 MPa with time dependency while decreasing up to 70% at 400–600 MPa. Some studies indicated the lactose influencing casein on HP, with 10% lactose addition in case in suspension before exposing it to 400 MPa for 40 min prevents the formation of large casein micelles. Number of researches has shown that moderate pressures (up to 400 MPa) and mild heating can activate or stabilize milk enzymes. Pressures of 350–400 MPa for 100 min can boost the activity of milk enzymes by up to 140%. This comprehensive and critical review will benefit scientific researchers and industrial experts in the field of HPP treatment of milk and its effect on milk components.

**Graphical abstract:**

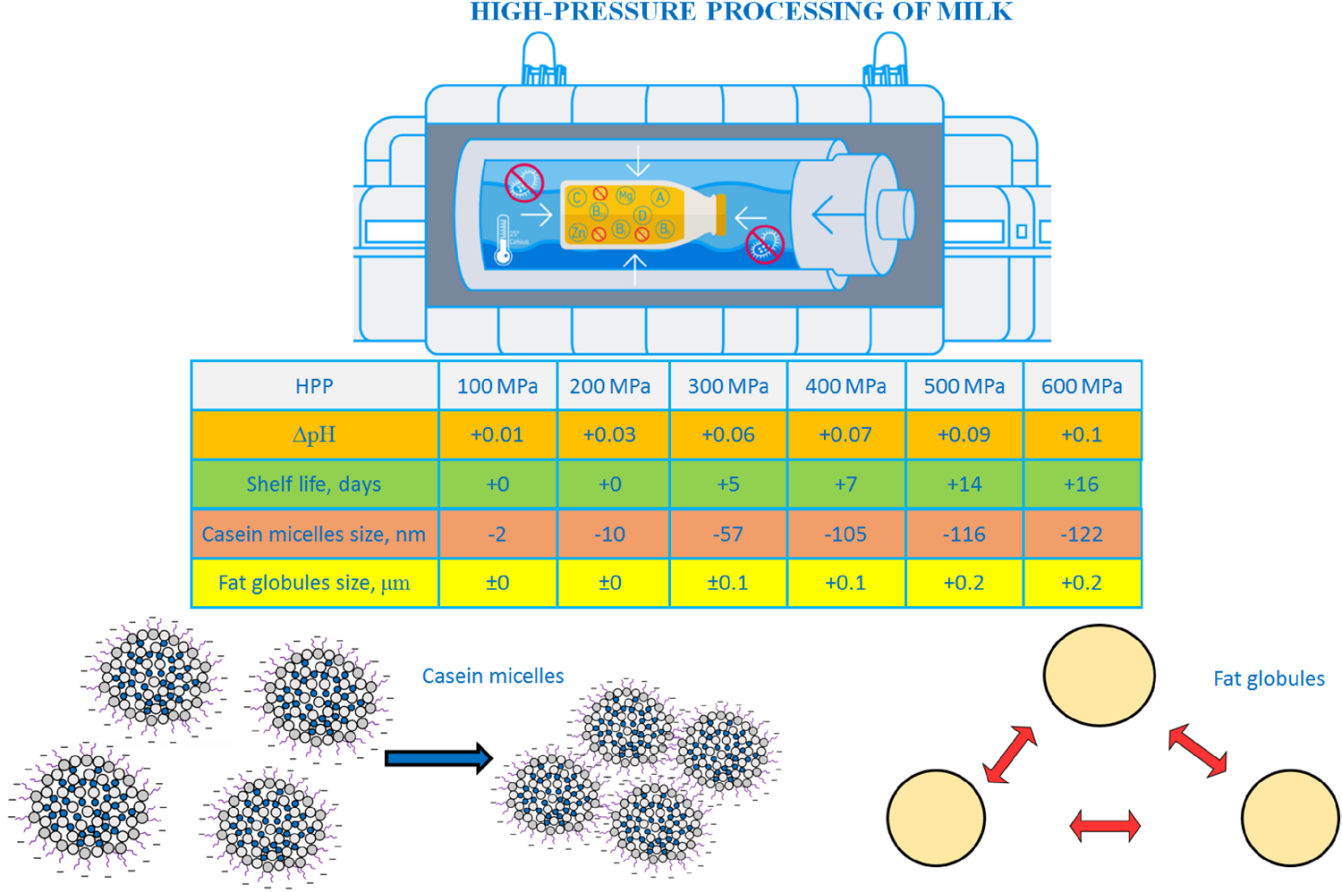

## Introduction

A plethora of research has been conducted on the impact of heat treatments on milk proteins during the last 60 years. Innovative and environmentally friendly dairy processing methods include ultrasound-assisted processing (UAP), microwave-assisted processing (MAP), and high-pressure processing (HPP). In general, novel technologies are less effective than traditional methods, so they are used in conjunction with fermentation and enzymatic hydrolysis, and are promising pretreatments to modify peptide profiles, improve yields, and increase bioactive peptide liberation when compared to conventional technologies (Murtaza et al. 2022). Although UAP is a unique and efficient technique because its mechanical effects and cavitation affect the protein structure, boost the biological activities of enzymes, and improve the rate of enzymatic hydrolysis (Garza-Cadena et al. 2023), HPP acts as a promising method of technological processing, which leads to some changes in the molecular structure of proteins and provides the appearance of new properties that cannot be achieved by using conventional methods of protein modification (Sergius-Ronot et al. 2022). HPP gives assurance on minimal changes in in milk quality, including organoleptic and rheological properties, and microbial safety on milk products (Ravash et al. 2022).

HPP on milk was initially reported by Hite (1899), which was only in the last few decades when HPP was researched for manufacturing of different dairy products with its objective of being an alternative to pasteurization. Some research studies have characterized the HPP-induced changes in the milk components using conventional methods of protein modification (Ramírez et al. 2021; Liang et al. 2023; Manin et al. 2023). The main thermodynamic approach to modifications caused by high hydrostatic pressure is based on the compressibility of molecules and changes in their volume (∆V) (Ni et al. 2021). Such physical impact leads to an equilibrium shift in favor of the state with the smallest total volume. Studies conducted so far indicate that HPP mainly breaks the non-covalent bonds including iconic and hydrophobic interactions while the covalent ones are not affected. For instance, HPP has a destructive effect on the quaternary (> 150 MPa) and tertiary (> 200 MPa) structure of most globular proteins, but causes a relatively small effect on the secondary structure (> 300–700 MPa). Protein denaturation includes dissociation of oligomeric proteins, unfolding and aggregation. The covalent bonds of the protein remain unaffected (Dubois et al. 2020). These changes depend on the structure and concentration of the protein, pressure, temperature, pH, ionic strength, composition solvent. Denaturation under pressure is an easily controlled process and causes less significant rearrangements in the protein globule than temperature or chemical denaturation. Therefore, proteins and other macronutrients can experience structural changes owing to HPP while the vitamins, flavor, color, and other small compounds remain practically intact (Leite Júnior et al. 2017). In dairy products, the action of HPP needs to be carefully analyzed to understand the impact on the bioactivity of components at different levels of pressure, time, temperature, microbial safety, etc. A combination of techniques are also being used and approved, including high temperature and HPP, namely, pressure-assisted thermal processing (PATP), that was approved by FDA in 2009 for commercial sterilization with the potential to replace ultra-high temperature (UHT) treatments (Sánchez et al. 2020).

**Fig. 1:**
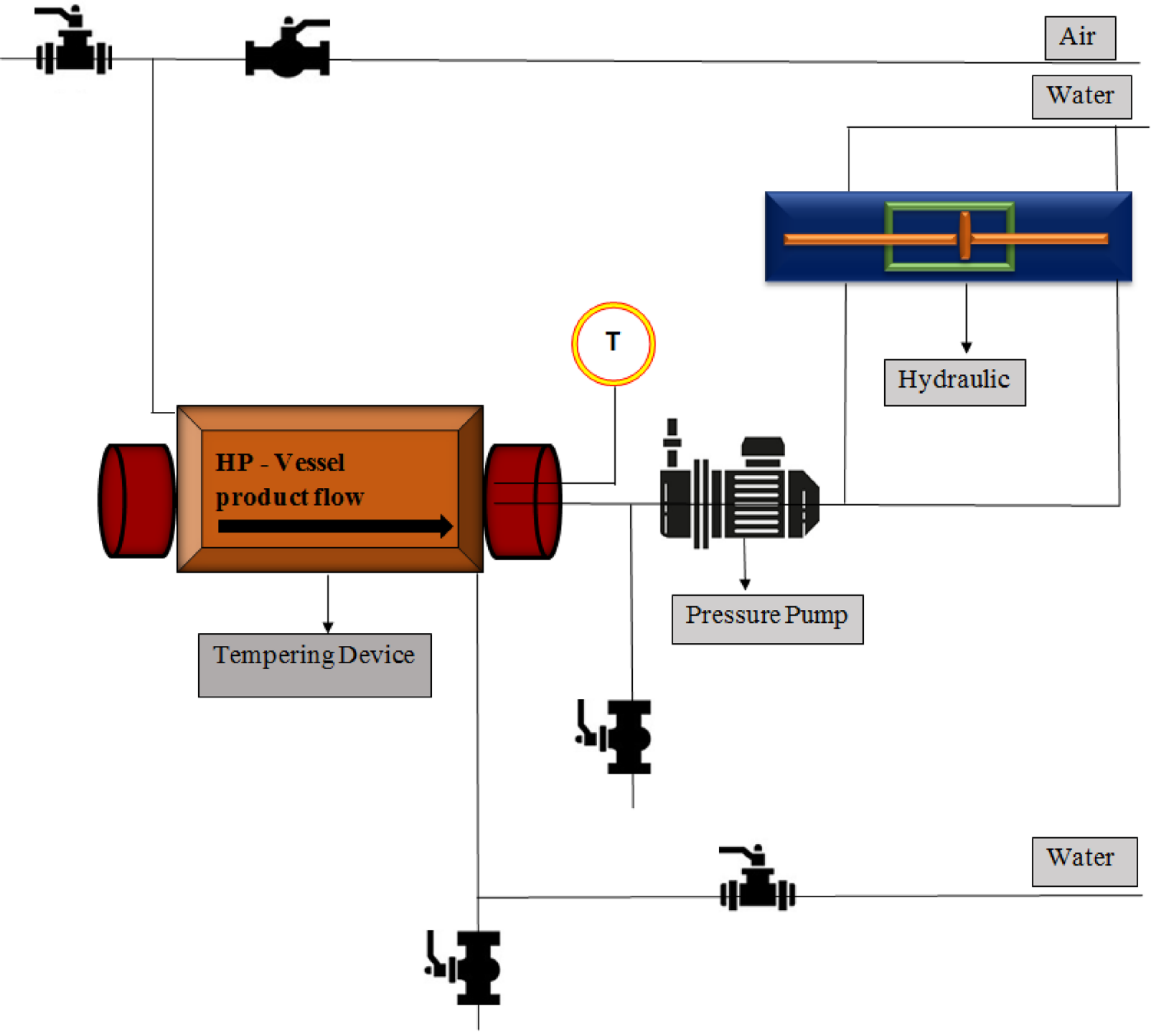
Working principles of HPP with specifications highlighted

The basic working principles of HPP is depicted in Fig. 1. HPP is a promising alternative to conventional thermal pasteurization with its ability to inactivate foodborne pathogens resulting in minimum nutritional losses along with maintaining fresh-like attributes of the food products. It is significantly efficient in eliminating vegetative microorganisms (Dhineshkumar et al. 2016). Similarly, it also influences the physicochemical and technological characteristics of milk components where pressure can impact the casein micelles along with whey protein structure. However, no impact was observed on the lactose content in milk suggesting no Millard reaction or lactose isomerization reaction in milk because of HPP (Stratakos et al. 2019).

**Fig. 2:**
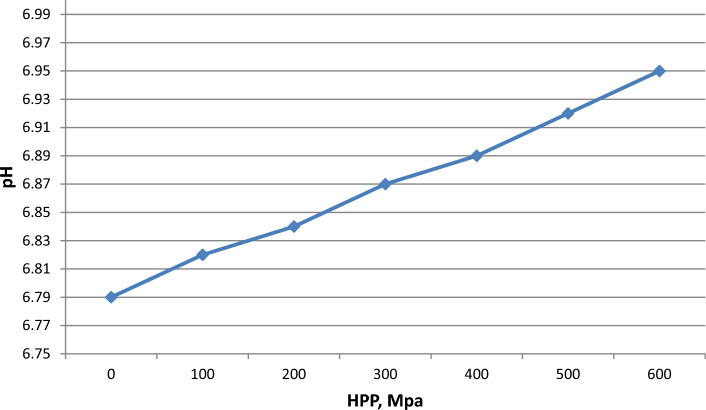
Effect of HPP on pH of raw milk (Tan et al. 2020; Stratakos et al. 2019; Serna-Hernandez et al. 2021)

Recent studies highlighted the effectivity of HPP (400–600 MPa and exposure times of 1–5 min) in reducing the *Escherichia coli*, *Salmonella,* and *L. monocytogenes* for up to 5 logs along with enhanced shelf life of raw milk by reduction of *Enterobacteriaceae*, *lactic acid bacteria*, *Pseudomonas* spp. The particle size, color, and mouthfeel of raw milk were also preserved as compared to pasteurized milk (Stratakos et al. 2019). Recent research also confirmed the role of HPP in enhancing the shelf life of goat milk and improving its overall quality and sensory attributes (Razali et al. 2021). Similar recent research also supported the same claim for cow milk where HPP enhanced the shelf life to 22 days when stored at 8 °C, without any changes in pH and no sign of microbial contamination. Similar results were observed for goat milk where slight change increase in pH (0.04%) was observed without any variations in compositional profile of the milk (Tan et al. 2020). In general, the researchers obtained reproducible results on the effect of HPP on the pH of raw milk. The generalized graph is shown in Fig. 2.

Thus, HPP slightly affects the pH of milk, increasing it by ∆pH = 0.1 when processing 600 MPa for at least 7 min. At the same time, as noted above, the pH of milk after HPP practically does not change during three weeks of storage.

## Impact of HPP on structure and function of main milk constituents

Tables 1 and 2 both shows the overview of HPP Effects on main milk constituents, discussed below. Table 1 focuses on the impacts of HPP on the principal proteins in milk, while Table 2 shows highlights of the HPP effects on the other components in milk, such as enzyme, milk fat globules. Other components, such as lipid, not affected by HPP are also discussed in this section.

**Table 1:** Overview of HPP Effects on caseins, β-lactoglobulin (β-lg), α-lactalbumin (α-la), and Immunoglobulin (*I*_g_)

HPP methods	Milk components	Concentration	HPP application	Effects	References
HPP	Micellar casein	2.5 and 10% (wt/vol)	150–450 MPa for 15 min	Increase of calcium-binding αS1- and αS2-casein levels in soluble phase Increase of soluble calcium and phosphorus levels with pressurization up to 350 MPa Reduction of levels of soluble Ca with treatment at 450 MPa	Cadesky et al. (2017)
HPLT	Micellar Caseins	2% (w/w)	100–600 MPa at pH 7.0 or 5.8 at − 15 °C, − 35 °C for 10 min	Formation of one hand large aggregates (flocs) Increase in solubility by creation of smaller micelles	Baier et al. (2015)
	Whey protein	2% (w/w)	100–600 MPa at pH 7.0 or 5.8 at − 15 °C, − 35 °C for 10 min	Without caseins, effects on solubility at pH 5.8 Without caseins, effects on solubility at pH 7	
(HHP)	Micellar casein	Concentration of 5% (w/w)	100 to 500 MPa for 5–20 min	Increase in free calcium in reconstituted solution with smaller micelle sizes Quickest dispersion process and best solubility with pressure 300 MPa	Ni et al. (2021)
UHPH	Casein micelles	–	100, 200, and 300 MPa for 10 min	Structural changes of the CN micellesAn increase of the average particle size at pH 8.5 No influence on average caseins micelle size at pH 6.7 and 8.5 Significant increase of protein content into the serum fractions at pH 10.5	Touhami et al. (2022)
HPP	Casein		60–120 MPa	More hydrophilic amino acid residues Worse surface hydrophobicity Ameliorated solubility	Han et al. (2020)
HPUP	Casein	1.0 mg/mL	60–120 MPa	Improvement in the stability	Han et al. (2020)
HHP	Micellar casein concentrates	4 and 8% w/w	300, 450, and 600 MPa for of 5 min	Increased pressures significantly reduce spoilage population Increase of acidity with pressure 450 or 600 MPa Effects on color (a loss of lightness and increase of blue/grey tonalities) by HPP treatments with	
HPP	β-lactoglobulin (β-lg)/κ-carrageenan mixed gels	16, 20, 24, 28, 32%, w/v	0.1–600 MPa for 30 min, 25 °C	Increase in water holding capacity and textural properties with increase of pressure levels Dominant effects on the hydrophobic interaction in mixed gels by HPP More compact and smoother network structure with higher pressure	Li et al. (2020)
HPP	β-lactoglobulin gels	20% (w/v)	0.1–600 MPa for 30 min, 25 °C	Increase in gel strength and textural properties with increase of protein concentration and pressure The highest strength at pH 5.0 forming gel Decrease in α-helix and increased of β-lg with random coil in gels with the increase of pressure Formation of regular and stable network of β-Lg gels with pressure 600 MPa	Li et al. (2018)
HPP	lysozyme and β-lactoglobulin	0.5 and 1.0 mg/ml	up to 600 MPa for periods of up to 30 min	The greatest effect at 600 MPa The most pressure resistant protein was lysozyme and The secondary structural level of lysozyme was only influenced using 300 MPa at both pH 7 and 3.7	Sousa et al. (2014)
HHP	β-lactoglobulin	8 wt% β-lg in water	600 MPa for 10 min at 20 °C	The structural flexibility favored its unfolding and a fast interfacial protein film formation A lower stability against high dilatational deformation due to a slow migration towards unoccupied interfacial area	Kieserling et al. (2021a)
High pressure–temperature treatment	β-lactoglobulin	25 mmol/L	100–600 MPa at the temperature of 20–60 °C for 5 min	Decrease in binding ability when the pressure increased from 0.1 to 200 MPa Increase with the increase in pressure from 200 to 400 MPa A gradual decrease until a pressure of 600 MPa Decrease in binding ability with an increase in pressure at 60 °C	Chen et al. (2022)
High hydrostatic pressure	β-lactoglobulin	0.1 wt% in water	up to 600 MPa for 10 min at 20 °C	An increase in surface hydrophobicity leading to loss in tertiary structure	Kieserling et al. (2021b)
HPP	β-lactoglobulin	20 mg/ml	550 MPa for 5 min	Pressure modifies positions of the major β-lactoglobulin epitopes	Kurpiewska et al. (2019)
HPP	α-lactalbumin (α-la) and pelargonidin-3-glucoside	5 × 10^−4^ M	100, 300 and 500 MPa	Increase in the quenching constants of α-lactalbumin at pH 7.4 and pH 8.0 Increase in the accessible fraction at pH 8.0, The fractions at pH 6.0 and pH 7.4 were increased without difference Binding site α-lactalbumin of was the typical binding site of calcium ion and not changed during the processing	Zou et al. (2019)
HHP	α-lactalbumin and bovine serum albumin	–	100–800 MPa and at 10–40 °C for 60 min	No denaturation at pressures up to 400 MPa, Increase in denaturation at over 500 MPa with increasing holding time at each pressure/temperature	Anema (2022)
HHP	α-lactalbumin and β-lactoglobulin	–	600 MPa for 5, 10, and 15 min	A drastic aggregation of β-lg with acidified whey compared with control whey	Marciniak et al. (2020)
HPP	α-lactalbumin		0, 20, 40, 60, 80, 100, and 120 MPa for 5–15 min	more α-la tyrosine residues lower α-helix and higher random coil contents in laccase-treated α-la with increasing pressure Increased in emulsifying and gel properties Increase in the crosslinking degree and functionality	Ma et al. (2020)
HHP	α-lactalbumin and β-lactogbulin	0 to 5 mg/mL	600 MPa for 5 min	Increase in purity of α-la up to 78% with a recovery of 88% for solution without casein Decrease in purity (∼71%) with casein, leading aggregation and co-precipitation upon acidification	Marciniak et al. (2019)
HHP	Bovine α-lactalbumin	5% (wt/vol) in distilled water	200 to 600 MPa, 25 to 55 °C, and from 5 to 15 min	Molten globules with differences in their surface hydrophobicity and secondary and tertiary structures a decrease in the α-helix content concomitant with an increase in β-strand content as the pressure increased at pH values of 3 No changes in molecular size due to HPP-induced aggregation	Rodiles-López et al. (2010)
HHP	α-lactalbumin	5% (w/v)	200, 400 and 600 MPa at 25, 40 and 55 °C for 5–15 min	A positive effect on solubility, foaming capacity, foam stability, emulsion activity index and emulsion stability of this protein at pH 7 with pressure 600 MPa and 55 °C for 10 min Increase in the foaming and emulsifying properties of the protein generally improved by treating its solution at high pressures Important changes attributed to solubility	Rodiles-López et al. (2008)
HPP	α-lactalbumin, lysozyme and myoglobin in the presence of β-lactoglobulin	1–24%, w/v	800 MPa for 5 min	No gel with pressure 800 MPa In the presence of β-lg (5%, w/v), Formation gel for α-la and LZM (each 15%, w/v) but not for Mb (15%, w/v) Gel-forming ability of a globular protein under high pressure	He et al. (2013)
HHPP	Milk protein concentrate	–	400–600 MPa at 5 and 10 min)	more protein denaturation and aggregation with heat treatments than HPP treatments Lactoferrin and α-lactalbumin are protein aggregates	Sergius-Ronot et al. (2022)
HHP	Bovine Serum Albumin	Protein concentrations (12, 25, 50 and 100 mg/mL),	levels (600, 700 and 800 MPa) and treatment times (15, 25 min)	Characteristic behavior of a pseudoplastic fluid Influence on rheological properties of the system depend on pressure level, treatment time, and protein concentration, Changes in viscosity and the shape of the curves of the viscosity as a function of the shear stress with increasing the pressure level Changes in the protein structure at pressure levels under 600 MPa by reducing denaturation temperature	de Maria et al. (2015)
TP or HPP	Immunoglobulin (Ig) and leukocyte	–	400 or 600 MPa for 3 or 6 min)	No changes at 400 MPa (for 3 or 6 min) Reduction in the original immunoglobulins levels contrast at 600 MPa	Contador et al. (2013)
HPP	Immunoglobulin G	–	300 MPa for up to 60 min or at 400 MPa for up to 30 min	Reduction in total native aerobic bacteria, *E. coli, Salmonella Dublin*, bovine herpesvirus type 1, and feline calicivirus populations in bovine colostrum at 300 MPa (30, 45, and 60 min) and 400 MPa (10, 15, and 20 min) No decrease occurred in *Mycobacterium avium ssp.* paratuberculosis Decrease in IgG content of colostrum at 400 MPa for 15 min during the calf trial Increase in colostrum viscosity at 400 MPa for 15 min, with 2 of 14 samples requiring dilution with water for calf feeding	Foster et al. (2016)
TP and HPP	Immunoglobulin content and lysozyme	–	200, 400 and 600 MPa for 2.5, 15 and 30 min	Maintenance of IgA and losses of IgM and IgG (21% for both) at 600 MPa for 2.5 min	Sousa et al. (2014)
HPP	Immunoglobulin A (IgA)	–	400, 500, or 600 MPa for 5 min at 12 °C	High-pressure processing at 400 MPa for 5 min at 12 °C maintains the immunological protective capacity associated with IgA antibodies	Ramírez et al. (2021)
HPLTP	Immunoglobulin (IgM, IgA and IgG)	–	200–800 MPa at low initial temperatures (between − 15, and 50 °C) and for 1 s (flash treatments)	No changes at low pressures in Igs Reduction in control levels at 800 MPa No effects on antioxidant activity at the processing conditions chosen	Ramírez et al. (2021)

*HPP* High pressure processing; *HPLTP* High pressure—low temperature processing; *HHP* High hydrostatic pressure; *UHPH* Ultra-high-pressure homogenization; *HPUP* High-pressure ultrasonic processing; *HPTT* High pressure–temperature treatment; *HHPP* High hydrostatic pressure pasteurization; *TP* Thermal pasteurisation

**Table 2:** Overview of HPP effects on lactoferrin (LF), enzymes, and milk fat globules

HPP methods	Milk components	Concentration	HPP application	Effects	References
HPP	Native lactoferrin		350, 500, 600 MPa for 8–10 min at < 10 °C	Decrease by 35% in lactoferrin concentration after holder pasteurisation (HoP), but not HPP No changes on the nutritional composition of milk, including lactoferrin	Pitino et al. (2022)
HPP	Bovine lactoferrin	1% w/v	300–700 MPa at pH 4.0–6.0 for 30–60 min	Modification on the tertiary structure of LF with increased intensity of HPP, leading to partial denaturation and aggregation of LF Increase in solubility, foaming and emulsifying properties of LF	He et al. (2016)
HPP	Chymosin, calf rennet, bovine rennet, porcine pepsin, and protease from *R. miehei*	Recombinant chymosin (10.0% w/v), calf rennet (10.0% w/v), bovine rennet (10.0% w/v), porcine pepsin (10.0% w/v), and protease from R. miehei (10.0% w/v),	Camel chymosin (212 MPa/5 min/10 °C), calf rennet (280 MPa/20 min/25 °C), bovine rennet (222 MPa/5 min/23 °C), and porcine pepsin (50 MPa/5 min/20 °C) and under inactivation conditions for all enzymes (600 MPa/10 min/25 °C) including the protease from *Rhizomucor miehei*	Activation conditions: Increase in intrinsic fluorescence of samples with high pepsin concentration (porcine pepsin and bovine rennet), Increase in surface hydrophobicity Changes in secondary structure of all enzymes Inactivation conditions: Increases in surface hydrophobicity and a reduction of intrinsic fluorescence Changes in secondary structure	Leite Júnior, et al. (2017)
UHPP	Angiotensin-converting enzyme inhibitory (ACEI) activity and quality of milk fermented with *Lactobacillus delbrueckii* QS306		0, 200, 300, 400, 500, or 600 MPa for 10–30 min	Increase in ACEI activity, apparent viscosity, concentrations of polypeptides and volatile aromatic substances, umami, and richness Reduction in bitterness and astringency Maintaining antioxidant properties Maintaining high level of ACEI activity and good quality during storage	Wu et al. (2022)
HPH	Milk fat globules (D32)		100 MPa at 4–60 °C for 5 min	Decrease in D32 Adsorption of casein at ≥ 4 °C, β-lactoglobulin, and α-lactalbumin in milk and buttermilk at ≥ 40 °C, ≥ 20 °C, and 60 °C respectively, on the surface of milk fat globules	Kiełczewska et al. (2021)

*HPP* High pressure processing; *UHPP* Ultra-high pressure processing; *HPH* High pressure homogenization

### Caseins

HPP has a significant impact on casein micelles. Electron microscopy was utilized in one of the first investigations to assess the size of casein micelles following HPP treatment (Ravash et al. 2020). Since then, numerous approaches, including laser granulometry, transmission electron microscopy, turbidimetry, and photon correlation spectroscopy, have been employed to identify changes in casein micelles during or after pressurization (He et al. 2016; Ravash et al. 2020; Blinov et al. 2022). The pressure-induced unfolding of casein causes an increase in the surface hydrophobicity of the casein globule, which leads to aggregation monomers. These changes are partially reversible at pressure < 150 MPa (Cadesky et al. 2017).

According to Ravash et al. (2020), HPP between 100 and 200 MPa at 20 °C for 30 min resulted in little or no changes in casein micelles but HPP of 250 MPa for > 15 min resulted in a considerable increase in casein micelles. Casein aggregation causes an increase in the average size of casein micelles. Regardless of time or temperature, applying pressures > 400 MPa reduced the average size of casein micelles by up to 50%. (Serna-Hernandez et al. 2021). HPP (200–500 MPa) was used to treat caprine milk, which decreased the size and enhanced the hydration of casein micelles (Nassar et al. 2019). Furthermore, research on goat milk preserved by microfiltration revealed that the size of casein micelles reduced at highpressure 300–500 MPa (Nassar et al. 2020). The particle size was reduced and agglomerated after treatment with reconstituted micellar casein concentrate at pressures ranging from 450 to 600 MPa (Iturmendi et al. 2020). Similarly, applying pressures > 500 MPa (for 15 min) decreased the size of reformed casein micelles by 42.5% (Hemar et al. 2020). Yang et al. (2020) employed one- and two-cycle HPP for whole and skim milk. Both treatments decreased the size of casein micelles, although the two-cycle treatment had a somewhat smaller impact.

Casein fraction dissolution also lowers the average size of casein structures (Blinov et al. 2021). This might be due to the dissolution of colloidal calcium phosphate or the breakdown of hydrophobic connections (Cavender and Kerr 2020). However, prolonged HPP or heating (to 80–85 °C) can reverse casein dissociation due to the disintegration of the quaternary and tertiary structure of the protein (Anema 2022). Furthermore, the interfering action of denatured β-lactoglobulin can hinder casein aggregation (Chen et al. 2022). HPP can cause significant changes in the quaternary (> 150 MPa) and tertiary (> 200 MPa) protein structures. However, it has no effect on secondary structures since hydrogenic bonds are resistant to HPP. This is because HPP has no effect on covalent bonds and influence mainlythe noncovalent bonds of casein (Sergius-Ronot et al. 2022). On the other hand, the study of the circular dichroism spectra of casein micelles treated at 900 MPa revealed no changes in the secondary structure; the destruction of the tertiary structure of the protein was found to be only 10% (Ravash et al. 2020).

Caseins differ in their content and conformation structure. At pressures > 400 MPa, they dissolve in the following order: αs2-casein, αs1-casein, k-casein, and β-casein. Although this order is connected to the quantity of serine phosphate left and may be due to its hydrophobic tendency (Serna-Hernandez et al. 2021).

### Whey proteins

#### β-lactoglobulin (ß-Lg)

Whey protein behavior under HPP is very significant for milk and dairy products. Several studies have examined the effect of HPP on whey proteins. Meng et al. (2017) found that when pressure increased, the quantity of non-casein nitrogen in milk serum dropped, implying denaturation and insolubilization of whey proteins. HPP has the greatest effect on ß-Lg. There are just two disulfide bonds and one free –SH group in ß-Lg (Bogahawaththa et al. 2017). As a result, it is less stiff than α-La, which contains four disulfide bonds. Treatment of raw milk at up to 100 MPa does not denature ß-Lg and it stays in its original monomer form (Liepa et al. 2017). When pressure exceeds 100 MPa, ß-Lg unfolds and the free—SH group is exposed, which may interact with k-casein or other unfolded ß-Lg molecules (Meng et al. 2017). It causes an increase in the size of casein micelles and a little aggregation of ß-Lg molecules.

HPP causes significant denaturation of ß-Lg, with denaturation reaching 70–80% following 400 MPa treatment (Liepa et al. 2017; Ravash et al. 2020). At 400–800 MPa, there is minimal additional denaturation of ß-Lg (Nassar et al. 2019). Renaturation happens in 1–2 days at 20–40 °C during storage. At lower temperatures (5 °C), reassociation does not occur because the energy of atoms is too low to establish hydrophobic and ionic connections. As a result, the strength of hydrophobic interactions is quite weak at low temperatures. Ravash et al. (2020) studied the impact of temperature and pressure on the denaturation of ß-Lg. The authors observed almost 100% denaturation at 300 MPa and 60 °C or at 400 MPa and 40 °C.

It unfolds and produces dimers via disulfide connections between 100 and 450 MPa. During storage, this change is reversible. It creates polymers via disulfide bonds at pressures ranging from 450 to 800 MPa, and the process is irreversible. No denaturation of ß-Lg was detected at pressures of 100 MPa, but the amount of denaturation increased at higher pressures, with a sudden and substantial rise between 300 and 400 MPa (Yang et al. 2018). At 800 MPa, almost 90% of the entire ß-Lg was denatured. The degree of α-La denaturation was substantially lower than that of ß-Lg; at 600 MPa, approximately 10% of the α-LA was denatured, while at 800 MPa, around 50% of the α -LA was denatured. The degree of HPP-induced denaturation of β-Lg and α-La in milk rises with holding time, temperature, and pH (Nassar et al. 2019).

#### α-lactalbumin (α-La)

α-La is a omponent of whey proteins in cow milk ranges from 1.2 to 1.5 g/L, and it is the second-largest component in the whey protein fraction by concentration (20%) after β-Lg. α-La has four intramolecular disulfide bonds and no free thiol groups, and it possesses the best described molten globule (MG) state, which is very stable and hence a favored model for researching protein folding (Marciniak et al. 2020). Because it possesses four disulfide connections, α-La is more resistant to denaturation under pressure. Denaturation of α-La begins only at pressures > 400 MPa. Because it lacks a free –SH group (Nassar et al. 2019), no transformation of monomers into disulfide-bonded aggregates was seen at HPP of 400–800 MPa (Ambrosi et al. 2016).

Because α-La contains no free thiol groups and only a minor fraction of the protein forms aggregates even at pressures as high as 1000 MPa, thiol-induced oligomerization of this protein at HPP can only be accomplished by the addition of low-molecular-weight reducing agents such as cysteine, 2-mercaptoethanol, or dithiothreitol (Sun et al. 2021). Small aggregates of α-La were found at 1000 MPa because to bonding between Cys 6- Cys 120, which was more vulnerable to cleavage due to its surroundings (Ravash et al. 2020). With increased holding duration, temperature, and pH of milk, the degree of HPP-induced denaturation of α-La and β-Lg rises (Liepa et al. 2017). Some α-La and ß-Lg were also observed to be linked tothe milk fat globule membrane in HPP-treated whole milk (Yang et al. 2018).

#### Bovine serum albumin (BSA)

BSA is a 582 amino acid polypeptide with 17 disulfide bridges and one free thiol group, Cys 34. The BSA structure is made up of 76% helix, 10% twists, 23% extended chain, and no ß-sheets. It is particularly resistant to pressure up to 400 MPa (Liepa et al. 2017), most likely owing to a huge number of disulfide bonds,.Denaturation happens at slower pace over 400 MPa pressure. Immunoglobulins can withstand pressures of up to 300 MPa. Immunoglobulins in caprine milk were resistant to pressures up to 300 MPa, but denaturation occurred at a rate of 35% following treatment at 500 MPa (Ravash et al. 2020).

When treated with 800 MPa, a significant effect on the secondary structure of BSA was shown, in contrast to β-lactoglobulin (Antonov et al. 2022). However, pressure-induced changes in the secondary structure were reversible. The presence of fifteen disulfide bonds in BSA prevents protein aggregation at a pressure of 1270 MPa (Anema et al. 2022). Although, at higher pressure polymerization can occur due to free thiol groups (Antonov et al. 2022).

#### Immunoglobulins

HPP tended to cause less harm to short RNA molecules, particularly piRNA-sized ones, which remained essentially intact. Wesolowska et al. (2019) indicated comparable effects on the quantity of immunoglobulins and other bioactive substances. MicroRNA readings, while being greatly reduced, were detectable after HPP in the experiment of Smyczynska et al. (2020), The authors suggested that exosomal sequestration protects microRNA against higher pressure but does not prevent heat destruction. The capacity of milk exosomes to reduce the adverse impact of HPP on microRNA appears to be another intriguing property of milk and should be studied in the future.

#### Lactoferrin (LF)

LF, an iron binding glycoprotein found in many mammalians external secretions, is known for its ability to bind and transport iron ions, as well as its antibacterial, anti-inflammatory, anti-tumoral, and immunomodulatory properties (Yang et al. 2018). LF, a known functional food component, is utilized in a broad range of products including infant formula, probiotics, supplementary tablets, pet food, and cosmetics, as well as a natural iron solubilizer in food (Li et al. 2019). However, just a few research have looked at how HPP affects the structural and functional features of LF. Franco et al. (2018) studied the impact of HPP (400, 500, and 650 MPa for 15 min at 20 °C) on the structure and immunoreactivity of LF and found that the antibacterial activity of LF may be sustained after 400 MPa, 15 min treatment. When LF was treated to HPP at pressures > 500 MPa, the structure of LF was altered (Ramos et al. 2015) investigated the impact of HPP (450–700 MPa at 20 °C) on LF denaturation in skim milk, whey, and phosphate buffer. They discovered that as pressure and holding time rose, the denatured fraction of LF increased, and protein denatured slower in the buffer and milk systems than in the whey system. Mayayo et al., (2014) investigated the effect of HPP (300–650 MPa at 20 °C) and heat treatment (65–90 °C) on LF immunoreactivity and estimated kinetic parameters for its denaturation process. The findings showed that HPP might be a viable alternative to thermal pasteurization in terms of native LF preservation.

#### Enzymes

HPP can either activate or deactivate milk enzymes, but can be no impact on the milk enzymes due to dependence on the pressure levels. The HPP technique processes dairy products,such as matured cheeses by activating or deactivating proteolytic and lipolytic enzymes. The HPP impact, on the other hand, is dependent on the pressure level and the process parameters. In this way, adding 400 MPa pressure to bovine milk (Munir et al. 2020) and 200–300 MPa pressure to ewe milk (Ávila et al. 2017) boosted proteolytic activity during cheese ripening. Other research has shown that moderate pressures (up to 400 MPa) and mild heating can activate (Leite Júnior et al. 2017) or stabilize (Medina-Meza et al. 2014) milk enzymes. According to Nivedita and Hilton (2018), milk enzymes vary in their sensitivity to high pressure. Lipase, xanthine oxidase and lactoperoxidase are resistant to pressures up to 400 MPa. Phosphohexoseisomerase, γ-glutamyl transferase and alkaline phosphatase in milk are partially inactivated at pressures > 350, 400 and 600 MPa respectively, and almost completely inactivated at ∼550, 630 and 800 MPa respectively.

HPP increased the coagulating activity of recombinant chymosin, calf rennet, adult bovine rennet, and porcine pepsin without changing their nonspecific action (Leite Júnior et al. 2019). However, there is a limit to the amount of pressure that can be applied to each enzyme before its activity is lost owing to denaturation caused by increased temperature due to pressure processing (Leite Júnior et al. 2019; Medina-Meza et al. 2014). In general, dairy enzymes are more resistant to HPP than to heat processing. Lactoperoxidase, for example, retains 50% of its original activity after 4 h HPP at 800 MPa at 25–60 ℃. (Leite Júnior et al. 2019). Similarly, lysozyme can withstand a pressure of 400 MPa for 30 min (Sousa et al. 2014). These findings are significant for HPP-treated dairy products because the antibacterial activity is sustained due to the presence of enzymes that are heat sensitive. Nonetheless, various enzymes have varying responses to HPP. Some of them are resistant to alkaline phosphatase in human body, which is stable at 800 MPa for 8 min, although pressures > 200 MPa can readily render acid phosphatase inert. Lipase activity is favorable in matured cheeses. Pressures of 350–400 MPa for 100 min can boost the activity of this enzyme by up to 140% under these circumstances (Martínez-Rodríguez et al. 2014). Plasmin activity in milk and its products, on the other hand, dropped by 75% at 20 °C for 30 min (Perinban et al. 2019) and by 87% at 400 MPa at 60 °C for 15 min (Ravash et al. 2020).

### Lipid constituents

Among the lipids, the following constituents are considered.

#### Milk fat globule

Milk fat remains as an emulsion owing to the presence of milk fat globule (MFG) as a complex moiety (Sánchez et al. 2020). This structure contains triglycerides surrounded by milk fat globule membrane (MFGM). This membrane is constituent of two-layered phospholipids with the internal monolayer near to the lipid core and an outer bilayer (Alberts et al. 2002). The membrane also encompasses the presence of different polar lipids, cholesterol molecules, proteins, and other minor constituents, with an average size of 0.1–9 µm in diameter. Only sheep milk is reported to have a decrease in this size without any MFGM disruption owing to HPP (Sánchez et al. 2020). Proteins react differently, where whey proteins are seen binding with MFGM proteins via interactions of sulfhydryl-disulfide interchanging process, that later impacts the denaturation (like β-Lg, α-La) of MFGM proteins at a high-pressure state. HPP at 500 MPa–15 min had no significant effect on anti-rotavirus activity in lactadherin, while 600 MPa–15 min combination decreased 60% of the overall activity. Similarly, bovine xanthine oxidase (Xod) also exhibited a diminishing 43%, 62 and 98% for 400, 500 and 600 MPa HPP for 15 min (Sánchez et al. 2020). Therefore, for formula milk enriched with MFGM, this technique can hinder the functional attributes of small constituents.

From technological perspective, it was observed that HPP can affect the rate of milk fat adhering as cream with increased results at 100–250 MPa with time dependency while decreasing up to 70% at 400–600 MPa. The results were explained with IgM aggregation on low pressures that escalated the cold agglutination resulting in avoiding the MFGs interactions. Altering flocculation was also observed owing to HPP, as at 400 MPa, an increase of MFG diameter was recorded at 15 min, while at 500 MPa, diameter increase was visible after 10 min but later decreased owing to the destabilization of clumps over the time. Therefore, with high zeta-potential values, more flocculation was observed as compared to coalescence. This was associated with IgM aggregation that produces particles with multiple binding sites for MFGs, making large clusters that can enhance the creaming phenomena (Kiełczewska et al. 2021).

#### Lipids

The lipid composition of milk constitutes on triglycerides, cholesterol, phospholipids. On HPP exposure, fatty acids sustained the treatment along with other minor constituents around 250–900 MPa for 5 min analysis. Lipids being most pressure sensitive are more prone to get influenced by HPP. This is the case of lipid oxidation, in which kinetics is accelerated in the presence of high hydrostatic pressure. There has been increasing focus on the response of lipid components to HPP, especially considering the deleterious outcomes that secondary products of oxidation have on the final product (Medina-Meza et al. 2014). Triglycerides melting temperature enhances > 10 °C, with an individual increase in 100 MPa pressure indicated a susceptibility towards crystallization in case of increased pressures (Medina-Meza et al. 2014). Similarly, the impact of HPP on the microbial concentration also was researched. Some studies revealed the protective impact of fat towards microorganisms, while others found no effect at all (Podolak et al. 2020; Sehrawat et al. 2021). Gram-negative bacteria are strongly impacted with change in pressure and temperature, microbial strains present and the animal species used for the extraction of the milk varieties (Sánchez et al. 2020).

### Lactose

Limited research is conducted to evaluate the impact of HPP on lactose content of milk. Milk treated with 100–400 MPa for 10–60 min at 25 °C showed no signs of Millard reaction or the lactulose formation. Some studies revealed the protective role of lactose for globular proteins (secondary structures) (Chen et al. 2019; Tang 2020). The mechanism responsible for this protective influence involves the transferase of water molecules to nonpolar residual content to the inner side of proteins. This effect can also stabilize whey protein isolates and the concentrated treated with HPP, especially for food with high functional value (Baier et al. 2015). Some studies indicate the lactose influencing casein on HPP, with 10% lactose addition in casein suspension before exposing it to 400 MPa–40 min prevented the formation of large casein micelles (Kelly and Meena 2022; Ma et al. 2024). Since lactose doesn’t enable to calcium and casein aggregate association and it also inhibits hydrophobic interactions among the micellar fragments during the treatment. The lactose has negative impacts on reduction in bacterial load. An *E. coli* suspension K12 in phosphate buffer with 1% casein or lactose showed less signs of growth in phosphate buffer then the growth in the whole milk (Stratakos et al. 2019).

### Other constituents

Other than nutritional and organoleptic properties, HPP is also used to effectively study the properties of volatile constituents lost during the heat-treated mechanisms. Aldehydes and methyl ketones are promoted at higher temperatures, while at high pressures accompanied with higher temperatures enhances the formation of aldehydes. HPP prevents the sulfur compounds formation, that is generally associated with cooked flavor of milk that consequently renders low consumer acceptability. Therefore, a higher sensory score with in-depth sensory analysis can further enhance the consumer’ likeability towards HPP-treated milk samples (Sánchez et al. 2020).

## Impact of HPP on production of bioactive constituents in milk

The biological activities of milk proteins have reportedly been impacted on applying different processing techniques. HPP application (500 MPa for 1 min) to whey protein isolate (WPI) before the digestion of enzyme pepsin and pancreatin escalated in respiratory epithelial cells (exposed to lipopolysaccharide) (Ali Redha et al. 2022). Similarly, hydrolysates from casein extracted via HPP 100 MPa for 1.0 h using different proteases including elastase, trypsin, thermolysin, savinase and flavourzyme. Also, it is known to increase the anti-inflammatory properties. Flavourzyme hydrolysates reduced nitric oxide and also suppressed the cytokines in Lipopolysaccharide (LPS)-stimulated macrophage cells, known for their pro-inflammatory role (Ambrosi et al. 2016).

In preserving the bioactives of human milk (2–6 lactation week), HPP at 200 and 400 MPa preserved the IgG (82.24%) while showing no alteration in adiponectin level (38.55%) as compared to raw milk. HPP preserved adipokines, growth factor, lactoferrin, IgG constituents as compared to holder pasteurization (Wesolowska et al. 2018). It is also known to preserve the protein activity using hydrogen bonds and protein’s secondary structure, as beta sheet is more pressure persistent than alpha helix. Pressure below 400 MPa makes the protein structure reversible owing to weak hydrogen bonds and *Van der Walls* forces. It is also known to preserve the IgA antibodies (1.4 g/L) with 88% decrease at 500 MPa and 69% at 600 MPa observed in the studies (Aceti et al. 2020).

## Influence of HPP on the milk protein allergenicity

Allergenic proteins of bovine milk are mainly α-casein and β-Lg. Studies indicate that HPP when applied to WPI and β-Lg by ELISA with reference to antibodies found in rabbits, egg yolk, applied HPP to WPI solution resulting in antigenicity of β-Lg on the increase in pressure, holding period and temperature (Kleber et al. 2004, 2007; Sánchez et al. 2020). Similar treatments enhanced the proteins reactivity along with its specific antibodies (HPP to β-Lg, 100–500 MPa at 25 °C). Variable results were obtained when IgE was used in case of cow’s milk allergic patients (Meng et al. 2017). In another study, combined pressure (600 MPa for 6 min), with heat treatment (50 °C) of β-Lg in terms of allergenicity was considered (Orcajo et al. 2015). At room temperature, no variations were observed but from 75 to 95 °C allergenicity was considerably decreased (Kurpiewska et al. 2019; Sánchez et al. 2020). These changes in β-Lg was associated with changes in tertiary structure resulting in antigenicity and (Ma et al. 2020; Rodiles-López et al. 2008), allergenicity (Bogahawaththa et al. 2017). Milk immunogenicity was also studied at 400, 500 or 600 MPa for 15 min with cellular model accompanied with human peripheral blood mononuclear cells and exhibiting cytokines variating concentrations. T helper (Th)1 and Th2 cytokines are needed to be balanced with an increase in pressure of 500 MPa, replenishing the immunogenic milk protein capacity at 600 MPa (Bogahawaththa et al. 2017).

## The impact of HPP on functional properties of milk compounds

HPP methods, such as high-pressure homogenization and high hydrostatic pressure, were found to have positive effects on functional properties of α-lactalbumin and casein (Han et al. 2020; Ma et al. 2020; Rodiles-López et al. 2008). High pressure treated milk compounds demonstrated increased emulsifying properties and foam formation. Han et al., (2020) shown that the foaming properties disappear for casein at pressure 60–80 MPa, the increasing treated pressures shown a foaming properties. High pressure (300–700 MPa) for 30–60 min was investigated for lactoferrin properties by He et al., (2016) observing that increasing pressure increase the foaming capacity of lactoferrin, but the highest was found at 400 MPa, while the lower pressure improve the solubility of the lactoferrin. Rodiles-López et al., (2008) investigated effects of temperature and pH with the method of high hydrostatic pressure and found significant effects on foaming capacity of β-LG for all pH values. The highest foam stability was found at 600 MPa, 40 °C, pH 9 and 5 min. These cases show different milk compounds have different optimum condition to reach optimal foaming capacity. Besides pressure, pH was another main contributing factor towards the foaming ability of milk compounds.

The emulsifying properties are also highly related to physical changes in milk’s emulsion, like milk. Protein plays in important role on emulsification process to generate a high homogeneity of emulsion in oil/water systems. HPP improved emulsion stability of casein in milk (Han et al. 2020). For casein 60–120 MPa of pressure is a relatively mild high-pressure to obtain the homogeneous emulsion. Emulsifying properties of α-LA by high pressure, including emulsion stability (ES) and emulsifying activity index (EAI) were also investigated by Rodiles-López et al., (2008). The studies also reflected decreased EAI and lower solubility of alpha LA at 400–600 MPa/55 ℃. Nonetheless, at the condition, no effects were found in ES because loss of solubility and aggregation by the HPP process. Similar results were obtained by Baier et al., (2015) in study of effect of HPP on technological and rheological properties of whey protein. HPP led to a decreased ES for emulsions from whey protein solutions independent from the treatment pH, while the foam stability was increased for these samples. For lactoferrin treated at pressure 400 MPa for 30 min, increase in emulsion stability was observed, but treatment with pressure more than 400 MPa decreased the stability (He et al. 2016). Besides the pressure treatment, the droplet size and pH also need to be considered due strong impact on ES (He et al. 2016).

## Available technologies to improve milk quality and safety

Improvement of milk products’ quality and safety have been reported since many years ago. New processing technologies are commonly used by Industry; millisecond technology (Myer et al. 2016), plasma activated water (Perinban et al. 2019; Widyaningrum et al. 2021), microfiltration (France et al. 2021), high pressure processing, and ultraviolet (UV) treatment for food surface, milk disinfection, and food preservation (Cappozzo et al. 2015) (Chawla et al. 2021; Delorme et al. 2020). These technologies are used to inactivate spoilage and pathogenic bacteria to improve the shelf life and safety of the raw milk. However, there are technologies influencing the quality and properties of the milk constituents, such as HPP, plasma activated water and microfiltration.

These technologies influence milk quality in different ways. HPP treatments are effective in inactivating vegetative bacteria but are ineffective against spores (Sánchez et al. 2020). In this case, combination with heat treatment can help to inactivate the spores. Besides, HPP treatments have significant effects on the milk components, such as lipid, protein, and salt, influencing the quality and properties of the milks (Anema 2022; Kieserling et al. 2021b). Increase in milk salts solubility can alter mineral balance and physical properties especially milk appearance, which significantly influenced by disintegration of casein micelles (Anema 2022). Microfiltration approach is very effective to remove the bacteria and spores from milk, but the effectiveness depends on membrane fouling and spore/bacteria concentration in raw milk (Martínez-Rodríguez et al. 2014). This approach includes by filtering the component of the milk than passing through the membrane pores (France et al. 2021). Due to filtration process with membrane, a possibility for milk components, such as large protein clusters, can be retained due to its bigger sizes than the membrane pores (Martínez-Rodríguez et al. 2014). Furthermore, another technology is plasma activated water, done by using plasma generated form ambient oxygen, carbon dioxide, nitrogen, air, and other gases, dissolved in water (Perinban et al. 2019; Widyaningrum et al. 2021). Low pH and reactive ions used in plasma activated water inactivate the microorganisms by oxidizing microbial cells structural components (Widyaningrum et al. 2021). On the other hand, the treatment exerts physical–chemical damage on the milk components, such as proteins and fats.

Millisecond technology and UV irradiation of food surfaces effectively inactivate the pathogenic and spoilage bacteria without influencing the milk constituents during processing. The millisecond technology rapidly pre heat raw milk under pressure, and then the milk is depressurized with rapid heating and continued rapid cooling inactivate the bacteria (Myer et al. 2016). While UV irradiation is applied to inactivate bacteria using UV light wavelengths with the range 100 to 400 nm (Cappozzo et al. 2015). No milk constituents effects are found because the treatments of this method is only applied and exposed to the surface of the milk products and is not penetrated into the milk constituents. The effect on milk constituents is not due to the lower penetration but because of no rise in temperature during inactivation treatment (Pendyala et al. 2022; Vashisht et al. 2022). However, validation and additional testing for several parameters are important for the efficacy to inactivate more thermally robust bacteria (Myer et al. 2016).

The technologies applied to improve the quality and safety of milk products gave advantages and disadvantages in terms of the microbial inactivation and the effects on milk components. Those dis- and advantages needs to be considered in the preservation of milk.

## In vivo studies in the preservation of processed bioactives in humans

The in vivo studies related to effects of HPP methods on bioactives preservation are limited. Wemelle et al. (2022a) investigated potential high hydrostatic pressure processing (HHPP) to replace holder pasteurization (HoP) for the human breast milk sterilization. Two hormones, e.g. milk apelin and glucagon-like peptide 1 (GLP-1), were found to be degraded by HoP, but HPPP was found to effectively preserve both hormones and increase glucose tolerance by acting on gut contractions in adult mice. Another study by Wemelle et al. (2022b) who assessed in vivo for antioxidant activity of donor human milk using HHPP or HoP treatment in mice, found that HHPP treatment retained vitamins to near-raw milk levels while decreasing H_2_O_2_ content. When compared to HoP treatment, HHPP for donor milk delivery stimulated antioxidant defenses and lowered certain inflammatory markers in the liver and ileum. HHPP treatment for donor milk may improve preterm infant nutrition and health. In general, both studies show the better preservation of the milk bioactive and antioxidant activities with HPP than treatment with higher temperatures. More specific studies are required to assess the preservation of other bioactive compounds, such as whey protein, lipid, etc.

## Conclusion

With the latest finding, HPP has contributed valuably towards the enhanced shelf life, novelty, textural properties, nutritional profile and sensory characteristics of different milk-based products and milk obtained from different sources. Recent studies highlighted the effectivity of HPP (400–600 MPa and exposure times of 1–5 min) in reducing the *E. coli*, *Salmonella,* and *L. monocytogenes* for up to 5 logs along with enhanced shelf life of raw milk by reduction of *Enterobacteriaceae*, *lactic acid bacteria*, *Pseudomonas* spp. The particle size, color, and mouthfeel of raw milk treated by HPP stay preserved compared to pasteurized milk. It is noted, that HPP slightly affects the pH of milk, increasing it by ∆pH = 0.1 when processing 600 MPa for at least 7 min. Analysis of modern scientific sources has shown that pressure affects the main components of milk: proteins, fat, lactose, biologically active substances. HPP lowers the average size of casein structures. It can affect the rate of milk fat adhering as cream with increased results at 100–250 MPa with time dependency while decreasing up to 70% at 400–600 MPa. On HPP exposure, fatty acids sustain the treatment along with other minor constituents around 250–900 MPa for 5 min analysis. Limited research has been conducted to evaluate the impact of HPP on lactose content of milk. Milk treated with 100–400 MPa for 10–60 min at 25 °C showed no signs of Maillard reaction or the lactulose formation. Number of researches has shown that moderate pressures (up to 400 MPa) and mild heating can activate or stabilize milk enzymes. The biological activities of milk proteins have reportedly been impacted owing to different processing methodologies applied to them. Anti-inflammatory and antioxidant potential escalated in respiratory epithelial cells (exposed to lipopolysaccharide) with 500 MPa for 1 min, HPP application to whey protein isolate before the digestion of enzyme pepsin and pancreatin. HPP improves the emulsification and emulsion stability of casein in milk.

Although HPP requires higher financial investment, this non-thermal treatment reduces the harmful impacts along with enhancing the functional profile with higher added values. Similarly, growing market demand is recorded for HPP with clean label characteristics. Additionally, implementation of such technologies should be deeply evaluated by the food industries not only for cost comparison for wide scale products but also for large scale adoption, aligning rules and regulations for intended food products. Consumers demand for nutritionally rich foodstuff; hence these consumer targets can help us understand the requirement of alternative treatments for better bioactive constituents’ profile.

## Data Availability

All raw data are available upon request from corresponding author.

## References

[CR1] Aceti A, Cavallarin L, Martini S, Giribaldi M, Vitali F, Ambretti S, Zambrini V, Corvaglia L (2020) Effect of alternative pasteurization techniques on human milk’s bioactive proteins. J Pediatr Gastroenterol Nutr 70(4):508–512. 10.1097/MPG.0000000000002598 31880664 10.1097/MPG.0000000000002598

[CR2] Alberts B, Johnson A, Lewis J, Raff M, Roberts K, Walter P (2002). The lipid bilayer. https://www.ncbi.nlm.nih.gov/books/NBK26871/

[CR3] Ali Redha A, Valizadenia H, Siddiqui SA, Maqsood S (2022) A state-of-art review on camel milk proteins as an emerging source of bioactive peptides with diverse nutraceutical properties. Food Chem 373:131444. 10.1016/J.FOODCHEM.2021.131444 34717085 10.1016/J.FOODCHEM.2021.131444

[CR4] Ambrosi V, Polenta G, Gonzalez C, Ferrari G, Maresca P (2016) High hydrostatic pressure assisted enzymatic hydrolysis of whey proteins. Innov Food Sci Emerg Technol 38:294–301. 10.1016/J.IFSET.2016.05.009 10.1016/J.IFSET.2016.05.009

[CR5] Anema SG (2022) Denaturation of α-lactalbumin and bovine serum albumin in pressure-treated reconstituted skim milk. Food Chem Adv 1:100002. 10.1016/J.FOCHA.2021.100002 10.1016/J.FOCHA.2021.100002

[CR6] Antonov YA, Moldenaers P, Cardinaels R (2022) Binding of lambda carrageenan to bovine serum albumin and non-equilibrium effects of complexation. Food Hydrocolloids 126:107321. 10.1016/J.FOODHYD.2021.107321 10.1016/J.FOODHYD.2021.107321

[CR7] Ávila M, Gómez-Torres N, Delgado D, Gaya P, Garde S (2017) Effect of high-pressure treatments on proteolysis, volatile compounds, texture, colour, and sensory characteristics of semi-hard raw ewe milk cheese. Food Res Int 100:595–602. 10.1016/J.FOODRES.2017.07.043 28873726 10.1016/J.FOODRES.2017.07.043

[CR8] Baier D, Schmitt C, Knorr D (2015) Effect of high pressure—low temperature processing on composition and colloidal stability of casein micelles and whey proteins. Int Dairy J 43:51–60. 10.1016/J.IDAIRYJ.2014.11.008 10.1016/J.IDAIRYJ.2014.11.008

[CR9] Blinov AV, Siddiqui SA, Nagdalian AA, Blinova AA, Gvozdenko AA, Raffa VV, Oboturova NP, Golik AB, Maglakelidze DG, Ibrahim SA (2021) Investigation of the influence of Zinc-containing compounds on the components of the colloidal phase of milk. Arab J Chem 14(7):103229. 10.1016/J.ARABJC.2021.103229 10.1016/J.ARABJC.2021.103229

[CR10] Blinov AV, Siddiqui SA, Blinova AA, Khramtsov AG, Oboturova NP, Nagdalian AA, Simonov AN, Ibrahim SA (2022) Analysis of the dispersed composition of milk using photon correlation spectroscopy. J Food Compos Anal 108:104414. 10.1016/J.JFCA.2022.104414 10.1016/J.JFCA.2022.104414

[CR11] Bogahawaththa D, Chandrapala J, Vasiljevic T (2017) Thermal denaturation of bovine immunoglobulin G and its association with other whey proteins. Food Hydrocolloids 72:350–357. 10.1016/J.FOODHYD.2017.06.017 10.1016/J.FOODHYD.2017.06.017

[CR12] Cadesky L, Walkling-Ribeiro M, Kriner KT, Karwe MV, Moraru CI (2017) Structural changes induced by high-pressure processing in micellar casein and milk protein concentrates. J Dairy Sci 100(9):7055–7070. 10.3168/JDS.2016-12072 28647329 10.3168/JDS.2016-12072

[CR13] Cappozzo JC, Koutchma T, Barnes G (2015) Chemical characterization of milk after treatment with thermal (HTST and UHT) and nonthermal (turbulent flow ultraviolet) processing technologies. J Dairy Sci 98(8):5068–5079. 10.3168/JDS.2014-9190 26026762 10.3168/JDS.2014-9190

[CR14] Cavender GA, Kerr WL (2020) Microfluidization of full-fat ice cream mixes: effects on rheology and microstructure. J Food Process Eng 43(2):e13350. 10.1111/JFPE.13350 10.1111/JFPE.13350

[CR15] Chawla A, Lobacz A, Tarapata J, Zulewska J (2021) UV light application as a mean for disinfection applied in the dairy industry. Appl Sci 11(16):7285. 10.3390/APP11167285 10.3390/APP11167285

[CR16] Chen X, Bhandari B, Zhou P (2019) Insight into the effect of glycerol on stability of globular proteins in high protein model system. Food Chem 278:780–785. 10.1016/J.FOODCHEM.2018.11.117 30583443 10.1016/J.FOODCHEM.2018.11.117

[CR17] Chen G, Wu C, Chen X, Yang Z, Yang H (2022) Studying the effects of high pressure–temperature treatment on the structure and immunoreactivity of β-lactoglobulin using experimental and computational methods. Food Chem 372:131226. 10.1016/J.FOODCHEM.2021.131226 34627095 10.1016/J.FOODCHEM.2021.131226

[CR18] Contador R, Delgado-Adámez J, Delgado FJ, Cava R, Ramírez R (2013) Effect of thermal pasteurisation or high pressure processing on immunoglobulin and leukocyte contents of human milk. Int Dairy J 32(1):1–5. 10.1016/J.IDAIRYJ.2013.03.006 10.1016/J.IDAIRYJ.2013.03.006

[CR19] de Maria S, Ferrari G, Maresca P (2015) Rheological characterization and modelling of high pressure processed Bovine Serum Albumin. J Food Eng 153:39–44. 10.1016/J.JFOODENG.2014.12.013 10.1016/J.JFOODENG.2014.12.013

[CR20] Delorme MM, Guimarães JT, Coutinho NM, Balthazar CF, Rocha RS, Silva R, Margalho LP, Pimentel TC, Silva MC, Freitas MQ, Granato D (2020) Ultraviolet radiation: an interesting technology to preserve quality and safety of milk and dairy foods. Trends Food Sci Technol 102:146–154. 10.1016/J.TIFS.2020.06.001 10.1016/J.TIFS.2020.06.001

[CR21] Dhineshkumar V, Ramasamy D, Siddharth M (2016) High pressure processing technology in dairy processing: a review. Asian J Dairy Food Res 35(2):87–95. 10.18805/AJDFR.V35I2.10718 10.18805/AJDFR.V35I2.10718

[CR22] Dubois C, Herrada I, Barthe P, Roumestand C (2020) Combining high-pressure perturbation with NMR spectroscopy for a structural and dynamical characterization of protein folding pathways. Molecules 25(23):5551. 10.3390/MOLECULES25235551 33256081 10.3390/MOLECULES25235551 PMC7731413

[CR23] Foster DM, Poulsen KP, Sylvester HJ, Jacob ME, Casulli KE, Farkas BE (2016) Effect of high-pressure processing of bovine colostrum on immunoglobulin G concentration, pathogens, viscosity, and transfer of passive immunity to calves. J Dairy Sci 99(11):8575–8588. 10.3168/JDS.2016-11204 27638265 10.3168/JDS.2016-11204

[CR24] France TC, Kelly AL, Crowley SV, O’Mahony JA (2021) Cold microfiltration as an enabler of sustainable dairy protein ingredient innovation. Foods 10(9):2091. 10.3390/FOODS10092091 34574201 10.3390/FOODS10092091 PMC8468473

[CR25] Franco I, Pérez MD, Conesa C, Calvo M, Sánchez L (2018) Effect of technological treatments on bovine lactoferrin: an overview. Food Res Int 106:173–182. 10.1016/J.FOODRES.2017.12.016 29579916 10.1016/J.FOODRES.2017.12.016

[CR94] Garza-Cadena M, Ortega-Rivera D et al (2023) A comprehensive review on Ginger (Zingiber officinale) as a potential source of nutraceuticals for food formulations: towards the polishing of gingerol and other present biomolecules Food Chem 413:135629. 10.1016/j.foodchem.2023.135629 36753787 10.1016/j.foodchem.2023.135629

[CR26] Han T, Wang M, Wang Y, Tang L (2020) Effects of high-pressure homogenization and ultrasonic treatment on the structure and characteristics of casein. LWT 130:109560. 10.1016/J.LWT.2020.109560 10.1016/J.LWT.2020.109560

[CR27] He JS, Mu TH, Guo X, Zhu S, Azuma N, Kanno C (2013) Comparison of the gel-forming ability and gel properties of α-lactalbumin, lysozyme and myoglobin in the presence of β-lactoglobulin under high pressure. Food Hydrocolloids 33(2):415–424. 10.1016/J.FOODHYD.2013.04.010 10.1016/J.FOODHYD.2013.04.010

[CR28] He X, Mao L, Gao Y, Yuan F (2016) Effects of high pressure processing on the structural and functional properties of bovine lactoferrin. Innov Food Sci Emerg Technol 38:221–230. 10.1016/J.IFSET.2016.10.014 10.1016/J.IFSET.2016.10.014

[CR29] Hemar Y, Xu C, Wu S, Ashokkumar M (2020) Size reduction of “reformed casein micelles” by high-power ultrasound and high hydrostatic pressure. Ultrason Sonochem 63:104929. 10.1016/J.ULTSONCH.2019.104929 31945573 10.1016/J.ULTSONCH.2019.104929

[CR30] Hite BH (1899) The effect of pressure in the preservation of milk: a preliminary report. West Virginia Agricultural and Forestry Experiment Station Bulletins 58:15–35

[CR31] Iturmendi N, García A, Galarza U, Barba C, Fernández T, Maté JI (2020) Influence of high hydrostatic pressure treatments on the physicochemical, microbiological and rheological properties of reconstituted micellar casein concentrates. Food Hydrocolloids 106:105880. 10.1016/J.FOODHYD.2020.105880 10.1016/J.FOODHYD.2020.105880

[CR32] Júnior BR, Tribst AA, Ribeiro LR, Cristianini M (2017) Biophysical evaluation of milk-clotting enzymes processed by high pressure. Food Res Int 97:116–122. 10.1016/J.FOODRES.2017.03.042 28578031 10.1016/J.FOODRES.2017.03.042

[CR33] Júnior BR, Tribst AA, Ribeiro LR, Cristianini M (2019) High pressure processing impacts on the hydrolytic profile of milk coagulants. Food Biosci 31:100449. 10.1016/J.FBIO.2019.100449 10.1016/J.FBIO.2019.100449

[CR34] Kelly AL, Meena GS (2022) Non-thermal treatment of milk: principles and purpose. Encyclop Dairy Sci 4:708–716. 10.1016/B978-0-12-818766-1.00056-8 10.1016/B978-0-12-818766-1.00056-8

[CR35] Kiełczewska K, Ambroziak K, Krzykowska D, Aljewicz M (2021) The effect of high-pressure homogenisation on the size of milk fat globules and MFGM composition in sweet buttermilk and milk. Int Dairy J 113:104898. 10.1016/J.IDAIRYJ.2020.104898 10.1016/J.IDAIRYJ.2020.104898

[CR36] Kieserling H, Alsmeier IM, Steffen-Heins A, Keppler JK, Sevenich R, Rauh C, Wagemans AM, Drusch S (2021a) Interfacial film formation and film stability of high hydrostatic pressure-treated β-lactoglobulin. Food Hydrocolloids 119:106746. 10.1016/J.FOODHYD.2021.106746 10.1016/J.FOODHYD.2021.106746

[CR37] Kieserling H, Giefer P, Uttinger MJ, Lautenbach V, Nguyen T, Sevenich R, Lübbert C, Rauh C, Peukert W, Fritsching U, Drusch S, Maria Wagemans A (2021b) Structure and adsorption behavior of high hydrostatic pressure-treated β-lactoglobulin. J Colloid Interface Sci 596:173–183. 10.1016/J.JCIS.2021.03.051 33839350 10.1016/J.JCIS.2021.03.051

[CR38] Kleber N, Krause I, Illgner S, Hinrichs J (2004) The antigenic response of β-lactoglobulin is modulated by thermally induced aggregation. Eur Food Res Technol 219(2):105–110. 10.1007/S00217-004-0924-3/FIGURES/5 10.1007/S00217-004-0924-3/FIGURES/5

[CR39] Kleber N, Maier S, Hinrichs J (2007) Antigenic response of bovine β-lactoglobulin influenced by ultra-high pressure treatment and temperature. Innov Food Sci Emerg Technol 8(1):39–45. 10.1016/J.IFSET.2006.05.001 10.1016/J.IFSET.2006.05.001

[CR40] Kurpiewska K, Biela A, Loch JI, Lipowska J, Siuda M, Lewiński K (2019) Towards understanding the effect of high pressure on food protein allergenicity: β-lactoglobulin structural studies. Food Chem 270:315–321. 10.1016/J.FOODCHEM.2018.07.104 30174052 10.1016/J.FOODCHEM.2018.07.104

[CR41] Li X, Mao L, He X, Ma P, Gao Y, Yuan F (2018) Characterization of β-lactoglobulin gels induced by high pressure processing. Innov Food Sci Emerg Technol 47:335–345. 10.1016/J.IFSET.2018.03.022 10.1016/J.IFSET.2018.03.022

[CR42] Li Q, Lan H, Zhao Z (2019) Protection effect of sodium alginate against heat-induced structural changes of lactoferrin molecules at neutral pH. LWT 99:513–518. 10.1016/J.LWT.2018.10.019 10.1016/J.LWT.2018.10.019

[CR43] Li X, He X, Mao L, Gao Y, Yuan F (2020) Modification of the structural and rheological properties of β-lactoglobulin/κ-carrageenan mixed gels induced by high pressure processing. J Food Eng 274:109851. 10.1016/J.JFOODENG.2019.109851 10.1016/J.JFOODENG.2019.109851

[CR44] Liang N, Mohamed HM, Kim BJ, Burroughs S, Lowder A, Waite-Cusic J, Dallas DC (2023) High-pressure processing of human milk: a balance between microbial inactivation and bioactive protein preservation. J Nutr 153(9):2598–2611. 10.1016/j.tjnut.2023.07.001 37423385 10.1016/j.tjnut.2023.07.001 PMC10517232

[CR45] Liepa M, Zagorska J, Galoburda R (2017) Effect of high pressure processing on milk coagulation properties. Res Rural Dev 1:223–229. 10.22616/rrd.23.2017.033 10.22616/rrd.23.2017.033

[CR46] Ma L, Li A, Li T, Li M, Wang X, Hussain MA, Qayum A, Jiang Z, Hou J (2020) Structure and characterization of laccase-crosslinked α-lactalbumin: impacts of high pressure homogenization pretreatment. LWT 118:108843. 10.1016/J.LWT.2019.108843 10.1016/J.LWT.2019.108843

[CR47] Ma X, Feng R, Ahrné L, Orlien V (2024) Pressure-induced gelation of blended milk and pea protein suspensions. Food Hydrocolloids 146:109284. 10.1016/J.FOODHYD.2023.109284 10.1016/J.FOODHYD.2023.109284

[CR48] Manin LP, Rydlewski AA, Pizzo JS, da Cruz VHM, da Silva Alves E, Santos PDS, Mikcha JMG, Cristianini M, Santos OO, Visentainer JV (2023) Effects of pasteurization and high-pressure processing on the fatty acids, triacylglycerol profile, Dornic acidity, and macronutrients in mature human milk. J Food Compos Anal 115:104918. 10.1016/J.JFCA.2022.104918 10.1016/J.JFCA.2022.104918

[CR49] Marciniak A, Suwal S, Brisson G, Britten M, Pouliot Y, Doyen A (2019) Evaluation of casein as a binding ligand protein for purification of alpha-lactalbumin from beta-lactoglobulin under high hydrostatic pressure. Food Chem 275:193–196. 10.1016/J.FOODCHEM.2018.09.110 30724187 10.1016/J.FOODCHEM.2018.09.110

[CR50] Marciniak A, Suwal S, Touhami S, Chamberland J, Pouliot Y, Doyen A (2020) Production of highly purified fractions of α-lactalbumin and β-lactoglobulin from cheese whey using high hydrostatic pressure. J Dairy Sci 103(9):7939–7950. 10.3168/JDS.2019-17817 32622608 10.3168/JDS.2019-17817

[CR51] Martínez-Rodríguez Y, Acosta-Muñiz C, Olivas GI, Guerrero-Beltrán J, Rodrigo-Aliaga D, Mujica-Paz H, Welti-Chanes J, Sepulveda DR (2014) Effect of high hydrostatic pressure on mycelial development, spore viability and enzyme activity of Penicillium Roqueforti. Int J Food Microbiol 168–169:42–46. 10.1016/J.IJFOODMICRO.2013.10.012 24239974 10.1016/J.IJFOODMICRO.2013.10.012

[CR52] Mayayo C, Montserrat M, Ramos SJ, Martínez-Lorenzo MJ, Calvo M, Sánchez L, Pérez MD (2014) Kinetic parameters for high-pressure-induced denaturation of lactoferrin in human milk. Int Dairy J 39(2):246–252. 10.1016/J.IDAIRYJ.2014.07.001 10.1016/J.IDAIRYJ.2014.07.001

[CR53] Medina-Meza IG, Barnaba C, Barbosa-Cánovas GV (2014) Effects of high pressure processing on lipid oxidation: a review. Innov Food Sci Emerg Technol 22:1–10. 10.1016/J.IFSET.2013.10.012 10.1016/J.IFSET.2013.10.012

[CR54] Meng X, Bai Y, Gao J, Li X, Chen H (2017) Effects of high hydrostatic pressure on the structure and potential allergenicity of the major allergen bovine β-lactoglobulin. Food Chem 219:290–296. 10.1016/J.FOODCHEM.2016.09.153 27765229 10.1016/J.FOODCHEM.2016.09.153

[CR55] Munir M, Nadeem M, Mahmood Qureshi T, Gamlath CJ, Martin GJO, Hemar Y, Ashokkumar M (2020) Effect of sonication, microwaves and high-pressure processing on ACE-inhibitory activity and antioxidant potential of Cheddar cheese during ripening. Ultrason Sonochem 67:105140. 10.1016/J.ULTSONCH.2020.105140 32388000 10.1016/J.ULTSONCH.2020.105140

[CR56] Murtaza MA, Irfan S, Hafiz I, Ranjha MMAN, Rahaman A, Murtaza MS, Ibrahim SA, Siddiqui SA (2022) Conventional and novel technologies in the production of dairy bioactive peptides. Front Nutr 9:780151. 10.3389/FNUT.2022.780151/BIBTEX 35694165 10.3389/FNUT.2022.780151/BIBTEX PMC9178506

[CR57] Myer PR, Parker KR, Kanach AT, Zhu T, Morgan MT, Applegate BM (2016) The effect of a novel low temperature-short time (LTST) process to extend the shelf-life of fluid milk. Springerplus 5(1):1–12. 10.1186/S40064-016-2250-1/FIGURES/3 27350902 10.1186/S40064-016-2250-1/FIGURES/3 PMC4899401

[CR58] Nassar KS, Zhang S, Lu J, Pang X, Ragab ES, Yue Y, Lv J (2019) Combined effects of high-pressure treatment and storage temperature on the physicochemical properties of caprine milk. Int Dairy J 96:66–72. 10.1016/J.IDAIRYJ.2019.03.003 10.1016/J.IDAIRYJ.2019.03.003

[CR59] Nassar KS, Lu J, Pang X, Ragab ES, Yue Y, Zhang S, Lv J (2020) Rheological and microstructural properties of rennet gel made from caprine milk treated by HP. J Food Eng 267:109710. 10.1016/J.JFOODENG.2019.109710 10.1016/J.JFOODENG.2019.109710

[CR60] Ni D, Liao M, Ma L, Chen F, Liao X, Hu X, Miao S, Fitzpatrick J, Ji J (2021) Enhanced rehydration behaviors of micellar casein powder: The effects of high hydrostatic pressure treatments on micelle structures. Food Res Int 150:110797. 10.1016/J.FOODRES.2021.110797 34865812 10.1016/J.FOODRES.2021.110797

[CR61] Orcajo J, de Martinez Marañon I, de Marañon M (2015) Antigenic response of bovine β‐lactoglobulin influenced by ultra‐high pressure treatment in combination with high temperature. Clinical Transl Allergy. 10.1186/2045-7022-5-S3-P49 10.1186/2045-7022-5-S3-P49

[CR62] Pendyala B, Patras A, Gopisetty VVS, Vashisht P, Ravi R (2022) Inactivation of B. cereus spores in whole milk and almond milk by serpentine path coiled tube UV-C system: numerical simulation of flow field, lipid peroxidation and volatiles analysis. Food Res Int 160:111652. 10.1016/j.foodres.2022.111652 36076441 10.1016/j.foodres.2022.111652

[CR63] Perinban S, Orsat V, Raghavan V (2019) Nonthermal plasma-liquid interactions in food processing: a review. Compr Rev Food Sci Food Safety 18(6):1985–2008. 10.1111/1541-4337.12503 10.1111/1541-4337.12503 33336960

[CR64] Pitino MA, Unger S, Gill A, McGeer AJ, Doyen A, Pouliot Y, Bazinet RP, Kothari A, Mazzulli T, Stone D, O’Connor DL (2022) High pressure processing inactivates human cytomegalovirus and hepatitis A virus while preserving macronutrients and native lactoferrin in human milk. Innov Food Sci Emerg Technol 75:102891. 10.1016/J.IFSET.2021.102891 10.1016/J.IFSET.2021.102891

[CR65] Podolak R, Whitman D, Black DG (2020) Factors affecting microbial inactivation during high pressure processing in juices and beverages: a review. J Food Prot 83(9):1561–1575. 10.4315/JFP-20-096 32866244 10.4315/JFP-20-096

[CR66] Ramírez R, Garrido M, Rocha-Pimienta J, García-Parra J, Delgado-Adámez J (2021) Immunological components and antioxidant activity in human milk processed by different high pressure-thermal treatments at low initial temperature and flash holding times. Food Chem 343:128546. 10.1016/J.FOODCHEM.2020.128546 33214041 10.1016/J.FOODCHEM.2020.128546

[CR67] Ramos SJ, Chiquirrín M, García S, Condón S, Pérez MD (2015) Effect of high pressure treatment on inactivation of vegetative pathogens and on denaturation of whey proteins in different media. LWT Food Sci Technol 63(1):732–738. 10.1016/J.LWT.2015.03.085 10.1016/J.LWT.2015.03.085

[CR68] Ravash N, Peighambardoust SH, Soltanzadeh M, Pateiro M, Lorenzo JM (2020) Impact of high-pressure treatment on casein micelles, whey proteins, fat globules and enzymes activity in dairy products: a review. Crit Rev Food Sci Nutrit 62(11):2888–2908. 10.1080/10408398.2020.1860899 33345590 10.1080/10408398.2020.1860899

[CR69] Ravash N, Peighambardoust SH, Soltanzadeh M, Pateiro M, Lorenzo JM (2022) Impact of high-pressure treatment on casein micelles, whey proteins, fat globules and enzymes activity in dairy products: a review. Crit Rev Food Sci Nutr 62(11):2888–2908. 10.1080/10408398.2020.1860899 33345590 10.1080/10408398.2020.1860899

[CR70] Razali MF, Narayanan S, Hazmi NA, Abdul Karim Shah NN, Mustapa Kamal SM, Mohd Fauzi NA, Sulaiman A (2021) Minimal processing for goat milk preservation: effect of high-pressure processing on its quality. J Food Process Preserv 45(7):e15590. 10.1111/JFPP.15590 10.1111/JFPP.15590

[CR71] Rodiles-López JO, Jaramillo-Flores ME, Gutiérrez-López GF, Hernández-Arana A, Fosado-Quiroz RE (2008) Effect of high hydrostatic pressure on bovine α-lactalbumin functional properties. J Food Eng 87(3):363–370. 10.1016/J.JFOODENG.2007.12.014 10.1016/J.JFOODENG.2007.12.014

[CR72] Rodiles-López JO, Arroyo-Maya IJ, Jaramillo-Flores ME, Gutierrez-Lopez GF, Hernández-Arana AN, Barbosa-Cánovas GV, Niranjan K, Hernandez-Sanchez H (2010) Effects of high hydrostatic pressure on the structure of bovine α-lactalbumin. J Dairy Sci 93(4):1420–1428. 10.3168/JDS.2009-2786 20338419 10.3168/JDS.2009-2786

[CR73] Sánchez L, Pérez MD, Parrón JA (2020) HPP in dairy products: Impact on quality and applications. Present and Future of High Pressure Processing: A Tool for Developing Innovative, Sustainable, Safe and Healthy Foods. Elsevier, USA, pp 245–272

[CR74] Sehrawat R, Kaur BP, Nema PK, Tewari S, Kumar L (2021) Microbial inactivation by high pressure processing: principle, mechanism and factors responsible. Food Sci Biotechnol 30(1):19. 10.1007/S10068-020-00831-6 33552614 10.1007/S10068-020-00831-6 PMC7847475

[CR75] Sergius-Ronot M, Pitino MA, Suwal S, Shama S, Unger S, O’Connor DL, Pouliot Y, Doyen A (2022) Impact of holder, high temperature short time and high hydrostatic pressure pasteurization methods on protein structure and aggregation in a human milk protein concentrate. Food Chem 374:131808. 10.1016/J.FOODCHEM.2021.131808 35021581 10.1016/J.FOODCHEM.2021.131808

[CR76] Serna-Hernandez SO, Escobedo-Avellaneda Z, García-García R, Rostro-Alanis MD, Welti-Chanes J (2021) High hydrostatic pressure induced changes in the physicochemical and functional properties of milk and dairy products: a review. Foods 10(8):1867. 10.3390/FOODS10081867 34441644 10.3390/FOODS10081867 PMC8391368

[CR77] Smyczynska U, Bartlomiejczyk MA, Stanczak MM, Sztromwasser P, Wesolowska A, Barbarska O et al (2020) Impact of processing method on donated human breast milk microRNA content. PLoS ONE 15(7):e0236126. 10.1371/journal.pone.0236126 32667939 10.1371/journal.pone.0236126 PMC7363072

[CR78] Sousa SG, Santos MD, Fidalgo LG, Delgadillo I, Saraiva JA (2014) Effect of thermal pasteurisation and high-pressure processing on immunoglobulin content and lysozyme and lactoperoxidase activity in human colostrum. Food Chem 151:79–85. 10.1016/J.FOODCHEM.2013.11.024 24423505 10.1016/J.FOODCHEM.2013.11.024

[CR79] Stratakos AC, Inguglia ES, Linton M, Tollerton J, Murphy L, Corcionivoschi N, Koidis A, Tiwari BK (2019) Effect of high pressure processing on the safety, shelf life and quality of raw milk. Innov Food Sci Emerg Technol 52:325–333. 10.1016/J.IFSET.2019.01.009 10.1016/J.IFSET.2019.01.009

[CR80] Sun X, Chua JV, Le QA, Trujillo FJ, Oh M-H, Campbell DE, Mehr S, Lee NA (2021) A response surface methodology (RSM) approach for optimizing the attenuation of human IgE-reactivity to β-Lactoglobulin (β-Lg) by hydrostatic high pressure processing. Foods 10:1741. 10.3390/foods10081741 34441519 10.3390/foods10081741 PMC8394912

[CR81] Tan SF, Chin NL, Tee TP, Chooi SK (2020) Physico-chemical changes, microbiological properties, and storage shelf life of cow and goat milk from industrial high-pressure processing. Processes 8(6):697. 10.3390/pr8060697 10.3390/pr8060697

[CR82] Tang CH (2020) Globular proteins as soft particles for stabilizing emulsions: concepts and strategies. Food Hydrocolloids 103:105664. 10.1016/J.FOODHYD.2020.105664 10.1016/J.FOODHYD.2020.105664

[CR83] Touhami S, Marciniak A, Doyen A, Brisson G (2022) Effect of alkalinization and ultra-high-pressure homogenization on casein micelles in raw and pasteurized skim milk. J Dairy Sci. 10.3168/JDS.2021-20700 35086710 10.3168/JDS.2021-20700

[CR84] Vashisht P, Pendyala B, Patras A, Gopisetty VVS, Ravi R (2022) Design and efficiency evaluation of a mid-size serpentine Dean flow UV-C system for the processing of whole milk using computational fluid dynamics and biodosimetry. J Food Eng 335:111168. 10.1016/J.JFOODENG.2022.111168 10.1016/J.JFOODENG.2022.111168

[CR85] Wemelle E, Marousez L, Lesage J, De Lamballerie M, Knauf C, Carneiro L (2022a) In vivo assessment of antioxidant potential of human milk treated by holder pasteurization or high hydrostatic pressure processing: a preliminary study on intestinal and hepatic markers in adult mice. Antioxidants 11(6):1091. 10.3390/antiox11061091 35739988 10.3390/antiox11061091 PMC9220199

[CR86] Wemelle E, Marousez L, de Lamballerie M, Knauf C, Lesage J (2022b) High hydrostatic pressure processing of human milk increases Apelin and GLP-1 contents to modulate gut contraction and glucose metabolism in mice compared to holder pasteurization. Nutrients 14(1):219. 10.3390/nu14010219 35011094 10.3390/nu14010219 PMC8747192

[CR87] Wesolowska A, Sinkiewicz-Darol E, Barbarska O, Strom K, Rutkowska M, Karzel K, Rosiak E, Oledzka G, Orczyk-Pawilowicz M, Rzoska S, Borszewska-Kornacka MK (2018) New achievements in high-pressure processing to preserve human milk bioactivity. Front Pediatr 6:323. 10.3389/FPED.2018.00323/BIBTEX 30519550 10.3389/FPED.2018.00323/BIBTEX PMC6250976

[CR88] Wesolowska A, Sinkiewicz-Darol E, Barbarska O, Bernatowicz-Lojko U, Borszewska-Kornacka MK, van Goudoever JB (2019) Innovative techniques of processing human milk to preserve key components. Nutrients 11(5):1169. 10.3390/NU11051169 31137691 10.3390/NU11051169 PMC6566440

[CR89] Widyaningrum D, Sebastian C, Tota Pirdo K (2021) Application of non-thermal plasma for milk sterilization: a review. IOP Conf Series: Earth Environ Sci 794(1):012146. 10.1088/1755-1315/794/1/012146 10.1088/1755-1315/794/1/012146

[CR90] Wu N, Zhao Y, Wang Y, Shuang Q (2022) Effects of ultra-high pressure treatment on ACE inhibitory activity, antioxidant activity, and physicochemical properties of milk fermented with Lactobacillus delbrueckii QS306. J Dairy Sci. 10.3168/JDS.2021-20990 35094856 10.3168/JDS.2021-20990

[CR91] Yang Y, Zheng N, Zhao X, Yang J, Zhang Y, Han R, Qi Y, Zhao S, Li S, Wen F, Guo T, Zang C, Wang J (2018) Changes in bovine milk fat globule membrane proteins caused by heat procedures using a label-free proteomic approach. Food Res Int 113:1–8. 10.1016/J.FOODRES.2018.06.046 30195502 10.1016/J.FOODRES.2018.06.046

[CR92] Yang S, Liu G, Munk DME, Qin Z, Petersen MA, Cardoso DR, Otte J, Ahrné L (2020) Cycled high hydrostatic pressure processing of whole and skimmed milk: effects on physicochemical properties. Innov Food Sci Emerg Technol 63:102378. 10.1016/J.IFSET.2020.102378 10.1016/J.IFSET.2020.102378

[CR93] Zou H, Xu Z, Zhao L, Wang Y, Liao X (2019) Effects of high pressure processing on the interaction of α-lactalbumin and pelargonidin-3-glucoside. Food Chem 285:22–30. 10.1016/J.FOODCHEM.2019.01.129 30797338 10.1016/J.FOODCHEM.2019.01.129

